# Preparation of dental and nursing professionals within Swedish higher education: navigating to confidence in literacies and professional knowledge

**DOI:** 10.1186/s12909-024-06439-2

**Published:** 2024-12-10

**Authors:** Nikolaos Christidis, Jakob Tomasson, Armin Rataghi, Maria Christidis

**Affiliations:** 1 Division of Oral Rehabilitation, Department of Dental Medicine, Karolinska Institutet, Huddinge, SE-141 04 Sweden; 2 Department of Nursing, Sophiahemmet University, Stockholm, SE-114 86 Sweden; 3 Department of Neurobiology, Care Sciences and Society, Karolinska Institutet, Huddinge, SE-141 83 Sweden

**Keywords:** Professional literacy, Academic literacy, Professional knowledge, Dentistry, Nursing, Professional education, Confidence

## Abstract

**Background:**

The professional education of dentists and nurses includes literacy, academic and professional literacy, and professional knowledge. These have a reciprocal relationship and contribute to the development of students and professionals. However, this is an area in need of further exploration. Therefore, this study aimed to investigate dentists’ and nurses’ experiences of academic and professional literacy and professional knowledge at the time of their graduation, and five years into their profession.

**Methods:**

The material consisted of an evaluation distributed twice to dentists and nurses. The first time was immediately after graduation (degree evaluation), and the second time was five years after graduation (alum evaluation). Approximately 30% of the dentists and the nurses responded both times. We analyzed the data using non-parametrical methods.

**Results:**

Upon graduation, the dentists scored high in academic and professional literacy and knowledge. Five years into the profession, dentists reported experiencing challenges with communication in English and tasks related to equitable treatment and equal rights. Nurses followed a similar pattern as the dentists. Still, by graduation, the newly graduated nurses expressed concerns about communication in English, and promoting sustainable development within their profession. The challenges persisted five years into their profession, particularly in areas such as communication in English and sustainable development, as well as work related to equitable treatment and equal rights.

**Conclusions:**

The level of confidence and perception of a sufficient degree of knowledge regarding academic literacy, professional literacy, and professional knowledge is higher at the time of graduation in both professions compared to five years into the profession, where there is a decrease in areas concerning work related to equitability, and equality, and for dentists also in communication in English. Furthermore, nurses had a constant low confidence rating in both evaluations concerning sustainable development and communication in English. This indicates that targeted and continuous professional development is crucial to address these challenges and to bridge the gap between the knowledge and confidence levels at graduation and the evolving demands of professional practice over time. Thus, when reconstructing the overall curriculum in professional educations it is of great importance to provide tools to enhance future professional development rather than the perception of that they can rely solely on their education at graduation time.

## Introduction

### The interplay of literacy and professional knowledge

Literacy and professional knowledge have a reciprocal relationship, where literacy enables the learning of professional knowledge and becomes part of it. In this context, professional knowledge refers to the specialized knowledge, skills, and competencies required to perform effectively in a particular field, such as dentistry or nursing. It encompasses not only technical skills but also the judgement, communication, and ethical awareness essential for professional practice [1, 2]. Literacy is a concept that is related to social practice and the socio-cultural context in which it is embedded [3]. It is linked to *what* people do, *when*, and *why* [4, 5], and involves specific terminology, text types, communication features and language styles [6]. Literacy is not singular, but plural [4], reflecting its many forms and functions in different settings.

Thus, literacy goes beyond just reading and writing; it is linked to specific contexts, each with its own rules and concepts. A specific understanding of each type of literacy is necessary to effectively navigate and engage in various life situations. Furthermore, a single context may involve multiple literacies, encompassing different ways of reading, writing, and communicating. For example, in fields like dental and nursing education, both academic literacy and professional literacy play crucial roles and often overlap [7]. Academic literacy equips students with the reading, writing and communication skills needed to succeed in their studies. Meanwhile, professional literacy prepares students to read, write and communicate effectively as professionals within their specific fields, focusing on the skills that are essential for their future careers [8]. Together, these literacies contribute to developing well-rounded professional knowledge.

In professional education, students are expected to develop and master both types of literacies for use in their future professions [6]. An important component of both academic and professional literacy is *critical thinking*, which involves abilities such as analysis, perspective-shifting, and self-reflection [9–11]. These skills are vital not only for students but also for professionals, like dentists and nurses, who need to make informed, reflective decisions in their practice. In this way, critical thinking supports professional knowledge as it enables practitioners to apply both academic and professional literacies in thoughtful, context-sensitive ways. Models such as Kolb’s Experiential Learning Theory provide a framework for blending theoretical education with practical training by emphasizing the integration of active experimentation and reflective observation into learning experiences [12]. Similarly, Miller’s Pyramid of Clinical Competence is widely used in medical education to guide the progression from theoretical knowledge (“knows”) to practical skills (“does”) through simulation and real-world application [13]. Thus, critical thinking becomes one of several foundational components of professional knowing, alongside academic and professional literacy, that this study seeks to explore.

### The role of literacy in professional education

The importance of literacy skills is also underscored by national learning outcomes for professional education in dentistry and nursing, as stated in the Swedish Higher Education Ordinance (SFS 1993:100; https://www.uhr.se/en/start/laws-and-regulations/Laws-and-regulations/The-Higher-Education-Ordinance/). Another aspect closely related to professional literacy is equity and inclusion. Equity in the workplace is essential in the Swedish Higher Education Ordinance, where national learning outcomes emphasise ethical sensitivity and professional competence (SFS 1993:100; https://www.uhr.se/en/start/laws-and-regulations/Laws-and-regulations/The-Higher-Education-Ordinance/). Addressing equity, fosters an inclusive environment that strengthens collaborative practice and enables diverse perspectives to enhance patient care [14]. Although students enter higher education with varying levels of academic experience, previous education does not guarantee that students are adequately prepared with the necessary knowledge and understanding [9]. The link between literacy – academic literacy or otherwise – and social practice within a socio-cultural context, gives each type of literacy a character that is specific to particular fields of study [3, 15, 16]. This highlights the plural nature of literacy, reinforcing that it takes diverse forms suited to different contexts.

Students in professional education face a dual challenge: they must develop and master both academic and professional literacy according to meet the requirements of their education and future profession. This need has been highlighted by previous research, such as studies on nursing students writing their degree projects [17–19] and dental students taking notes during classroom teaching and clinical practice [7, 20]. Degree projects, often requiring extensive research and writing on a field-relevant topic, serve as a key learning tool in fostering these literacies. For example, dental students frequently experience a shift from initial uncertainty in clinical settings to greater confidence as they begin to apply theoretical knowledge to real-world patient cases. Supporting this, one study showed that final-year dental students reported higher self-perceived confidence in performing essential dental procedures following curriculum changes emphasizing competencies [21]. Similarly, nursing students in clinical placements describe how initial apprehension when applying classroom concepts in patient care evolves into confidence as their literacy and professional knowledge align. This was also shown in a study demonstrating that nursing students’ self-confidence in clinical competencies improved significantly after participating in high-dose simulation clinical teaching models [22]. Together, these findings illustrate how academic and professional literacy development prepares students to navigate the complexities of their future professions with greater assurance.

### Bridging academic and professional literacies from education to healthcare practice

According to previous studies, certain aspects are particularly important for students’ development of academic literacy [17–19]. Nursing students, for example, recognize that writing a degree project requires adherence to an established style and structure, but they also need educational support to understand how to effectively use these conventions [18, 19]. This support may come from supervisors, peers, and even individuals outside the educational context. Thus, while working on the degree project is an individual task, it is also collaborative, involving feedback from others, discussions that encourage argumentation perspective-shifting, and comparisons of text [18]. The degree project serves as both a personal and systemic learning tool, reflecting the multifaceted growth students experience as they develop their academic and professional literacies [17]. For nursing students, writing the degree project not only enhances their ability to analyse and structure information but also builds their confidence in making evidence-based decisions. It was shown that nursing students perceived the degree project as a significant factor in fostering academic writing skills and critical thinking [23]. Similarly, dental students often report that the research and critical analysis required for their projects directly support their clinical judgment and patient interaction, fostering a sense of readiness for professional roles. This was highlighted that final-year dental students’ self-assessed confidence in general dentistry improved significantly through engagement in comprehensive care programs [24].

In this way, literacy skills developed in the degree project relate to professional knowledge, as students prepare for scenarios they will encounter in their future careers: engaging with patients, identifying physiological functions and pathological conditions, and understanding work organization and stakeholder dynamics, as well as the relationship between patient populations and common diseases. However, studies have shown that nursing students’ perceptions of the degree project’s relevance to their future profession vary [19]. Some nursing students perceive a discrepancy between writing the degree project and their future professional work, while others see a clear connection. This suggests that the relation between academic and professional literacy is sometimes evident for students, but not always. Previous studies also emphasize the strong relationship between literacy skills in professional education and professional knowledge, which ultimately contributes to the development of a well-prepared professional, such as a dentist or nurse [17–19, 25, 26].

Research on dental students has highlighted that they often recognize a dual purpose for teaching and examinations: academic achievement and professional application [7, 20]. For instance, studies show that students often supplement teacher-constructed material with additional notes and illustrations [7]. These notes are not only used for exams but are also saved – on the students’ own initiative – for future use in their careers as dentists. In one study, a case-based examination required students to document an authentic patient case using a structured template and scientific references to support their professional judgments [20]. While some students found it challenging to balance professional content with academic requirements, feedback from teachers helped them navigate these difficulties effectively. Such assessments reinforced the connection between education and professional practice, integrating academic literacy, professional literacy, and professional knowledge. Moreover, both dentistry and nursing professionals emphasize the critical importance of practical experience and interpersonal skills, often viewing them as equally, if not more, valuable than academic qualifications. For example, it has previously been shown that healthcare professionals, including nurses, rated communication, teamwork, and problem-solving skills developed during clinical experiences as essential for effective patient care [27]. Similarly, a recent review reports that dentists attribute their ability to manage complex patient interactions more to hands-on clinical training than to formal academic instruction. These findings suggest that while academic literacy lays the groundwork for professional expertise, practical experience and interpersonal skills are indispensable for navigating the complexities of real-world healthcare settings [28].

While these previous studies provide knowledge on dental and nursing students’ experience of literacies and professional expertise in their training, this study includes these experiences in dentists’ and nurses’ career development. Studies specifically examining literacy during the transition from professional education to professional practice – for instance, among dentists and nurses – are scarce, as is research on literacy in combination with professional knowledge. A previous study on engineers’ transition from education to profession, focusing on writing, found that educational programs often do not fully prepare students for the range of literacy-related situations that may occur in the profession [29]. This raises a critical question: How can professional education balance the development of academic and professional literacy to ensure healthcare practitioners are not only prepared for immediate practice but also adaptable to the evolving demands of interdisciplinary healthcare environments?

Understanding the relationship between literacy and professional knowledge in both educational and clinical settings is essential for assessing how effectively professional education prepares healthcare practitioners. Thus, this study focused on literacy skills critical to enabling dentists and nurses to meet the demands of complex, interdisciplinary healthcare roles and deliver high-quality patient care. These skills include written and verbal communication, critical analysis, evidence-based practice, and interprofessional collaboration. Specifically, this study aimed to investigate dentists’ and nurses’ experiences of academic and professional literacy, as well as professional knowledge, in relation to their graduation from professional education, and five years into their professional careers.

## Materials and methods

### The study context

The study context was professional education in dentistry and in nursing at Karolinska Institutet (KI), in Sweden. The participants in this project completed the study program in dentistry at KI (2TL13), comprising 300 ECTS (European Credit Transfer System) credits, equal to five years of full-time studies (https://utbildning.ki.se/student/tandlakarprogrammet/programoversikt-tandlakarprogrammet). This program encompassed odontological subjects, but also biomedicine, basic science, medicine, behavioral science, and clinical training on-site at the university clinic. The program awarded a degree of Master of Science in Dental Surgery. Now, there is a new study program in dentistry (2TL19) that was under construction and implemented during the autumn of 2019. The study program in nursing at KI (1SJ18) comprises 180 ECTS credits, equal to three years of full-time studies (https://utbildning.ki.se/student/sjukskoterske-programmet/sjukskoterskeprogrammet-campus). It includes the subjects of nursing, medicine, public health, and social behavioral science, and there is also workplace based clinical training. The completion awards the degree Bachelor of Science in Nursing. Both programs – dentistry, and nursing – include learning objectives with academic and professional literacy. Depending on the course content within the program, there may be more emphasis on one type of literacy. However, both types of literacies are usually present because assessment of students’ performance is part of the specific educational context.

### Object of analysis

Professional education in dentistry and in nursing is assessed through various evaluations. These have a specific order in the program: Course evaluations at the end of each course, degree evaluations at the end of the program, and alumni evaluations five years after graduation.

The purpose of these evaluations is to provide insights for course and program managers to support the development of courses and educational programs. By collecting feedback on how well students and professionals feel their education translates into practical application, the evaluations aim to identify patterns and areas for improvement within the curriculum. Group-level analysis reveals overarching trends, offering a broader perspective on the effectiveness of educational strategies. As graduates enter diverse healthcare contexts, each with unique challenges and opportunities, these insights help educators understand which aspects of the curriculum are most relevant, allowing them to refine educational offerings to better meet stakeholder needs.

In this study, the material comprised of the degree evaluation from dental and nursing students graduating from KI in June 2017, and the alum evaluation from the same students in June 2022. They were all from the same student cohorts (dentistry and nursing respectively).

### The degree evaluation

The degree evaluation comprised 46 questions on a Likert scale ranging from 1 to 6. A score of 1 indicates that the newly graduated student strongly disagrees and 6 indicates that the newly graduated student strongly agrees with the statement. All mean scores above 4 were considered good, i.e. that the respondents agreed with the question/statement.

There were twenty questions about the students’ study period at KI. For instance, the questions concerned whether they got enough feedback on assignments, seminars, and practical training for their coming profession, if the courses were based on current research, and if the physical premises of the different campuses were sufficient for the students’ needs to study.

There were also twenty questions about specific skills vital for work in different healthcare professions where the individual is responsible for the care of patients. For example, one question assessed confidence in using the English language, which is important not only for professional development – such as reading research articles and staying current with medical advancements – but also for communicating with patients whose first language is not Swedish. These questions were of special interest in this paper.

Four questions concerned equality. For instance, whether the former student felt well prepared for his/her coming work demands to act for equality, equal treatment based on ethnicity, religion, social class etc. Also, there were questions that concerned equal rights from an LGBTQIA + perspective (lesbian, gay, bisexual, transgender, queer/questioning –one’s sexual or gender identity – intersex, and asexual/aromantic/agender), and equal treatment of disabled individuals. This focus on equality is especially important in healthcare professions in Sweden, as it encompasses fair treatment and effective communication with patients and colleagues, regardless of their ethnic, cultural, sexual, or religious backgrounds. Moreover, it includes the ability to recognise and address ethical dilemmas that may arise in the workplace.

Also, the degree evaluation had two questions that concerned whether the former student is satisfied with their study period at KI overall and if they would recommend KI to new students.

### The alum evaluation

The alum evaluation comprised 58 questions on a Likert scale ranging from 1 to 6. A score of 1 indicates that the five-year experienced dentist/nurse strongly disagrees and 6 indicates that the five-year experienced dentist/nurse strongly agrees with the statement. All mean scores above 4 were considered good, i.e. that the respondents agreed with the question/statement.

The first third of the questions were about what program the former student studied at KI, what their current work is and if it is related to their education. The other 2/3 of the questions were about equality and specific work skills. The different skills were divided into two categories, one where the individual was asked how well their education corresponded to their current works demands, and the other to what extent their education contributed to develop knowledge and abilities in the different work skills that were asked about. Most of these skills were the same as asked for in the degree evaluation and were of special interest in this paper.

### Statistical analysis

The statistical analysis of the evaluations was performed with the statistical program *Jamovi* (*jamovi* (Version 2.3) [Computer Software], Sydney, Australia (retrieved from https://www.jamovi.org 2024-01-11). Data was tested for normality with the Shapiro-Wilk’s test. Parametrical statistical methods were used for data that was normally distributed. Non-parametrical statistical methods were used for data that was not normally distributed and on an ordinal scale. Since all data was on an ordinal scale the Wilcoxon-test was used to test differences between newly graduated students and professionals after five years of working experience. The respondents belonged to the same cohort responding to both evaluations. Further, the Wilcoxon test was used to test for differences between the two study programs. The significance level was set to *p* < 0.05.

## Results

### Dental student perception

Twenty three out of 71 (33%) newly graduated dental students answered the degree evaluation, while twelve out of these 71 students answered the alum evaluation that was distributed five years after graduation. At the time for their graduation, and five years after graduation, the dental students scored high on the Likert scale with mean scores ranging from 4.48 (for how well prepared they are for practicing their profession) to 5.61 (to collaborate with others). This indicates that the newly graduated dental students in general felt well prepared. However, five years in their profession this was almost the same with some scores being slightly lower, while others higher now ranging from 3.75 (to communicate in English) to the maximal possible 6.00 (to critically analyze information).

Even though there was a large decrease in mean score regarding how prepared the dental students were for their profession concerning communication in English (decreased from 5.22 to 3.75) this was not a significant decrease. There was, however one score that differed significantly between the newly graduated dental students and then five years in their profession. This concerned how prepared they were regarding equitable treatment based on ethnic background, religion, social class, etc. which decreased from 5.74 to 4.0, but still considered good (scores from 4 to 6). All the scores are presented in Table 1.

In summary, at the time of graduation and five years after their graduation, newly graduated dental students and dentists perceived that they had knowledge to find relevant or necessary information, to critically analyze information and to use it for an evidence-based practice. They also perceived that they had knowledge, skills/abilities, and tools to practice dentistry in all aspects and to collaborate. Except for English they also felt well prepared to communicate verbally and in writing in Swedish.

**Table 1:** Distribution of responses mean (SD) from newly graduated dental students (23/71) as well as after five years in their profession (12/71), from Karolinska Institutet, Sweden, regarding professional knowledge

	Newly graduated mean= (SD)	After 5 years in profession mean= (SD)	*p*-value
**I feel well prepared for my future profession needs**:			
To communicate in writing	5.52 (0.73)	5.00 (1.21)	0.202
To communicate verbally	5.57 (0.728)	5.58 (0.669)	1.0
To communicate in English	5.22 (1.17)	3.75 (1.14)	0.063
To critically analyze information	5.61 (0.656)	6.00 (0.9)	0.588
To find relevant/necessary information	5.57 (0.59)	5.17 (1.34)	0.59
To practice evidence based	5.48 (0.665)	5.75 (0.452)	0.120
To follow the development in my profession	5.13 (0.968)	5.00 (1.21)	1.000
To practice in my profession	4.48 (1.31)	5.00 (0.996)	0.276
To practice independently	5.39 (0.839)	4.18 (1.47)	0.05
To apply practical skills	5.35 (0.647)	5.58 (0.669)	0.219
To independently solve problems	5.17 (0.887)	5.50 (0.674)	0.154
To promote sustainable development	4.70 (1.40)	4.50 (1.62)	1.0
To collaborate with others	5.61 (0.656)	5.67 (0.651)	0.48
To collaborate with other professions	5.09 (1.12)	4.67 (0.985)	0.202
To work in interprofessional teams	4.83 (1.47)	5.42 (0.793)	0.078
To handle ethical considerations	5.48 (0.593)	5.50 (0.674)	0.608
To collaborate in multicultural environments	5.22 (1.20)	5.00 (1.21)	0.943
To work for equality	5.36 (1.36)	3.92 (1.73)	0.06
Equitable treatment based on ethnic background, religion, social class, etc.	5.74 (0.541)*	4 (2.09)	0.032
Equal rights from an LGBTQIA + perspective	5.43 (1.41)	3.08 (2.43)	0.051
Equitable treatment of people with disabilities	5.52 (1.31)	3.67 (2.31)	0.111

* = Indicates significant difference (*p* < 0.05; Wilcoxon-test)

### Nursing student perception

Fifty three out of 163 (33%) newly graduated nursing students answered the degree evaluation, and 32 out of these 163 students answered the alum evaluation five years after graduation.

At the time for their graduation, and five years after graduation, the nursing students scored, in general, high on the Likert scale with mean scores ranging from 3.66 (for how well prepared they were to communicate in English) to 5.45 (how prepared they were concerning equitable treatment based on ethnic background, religion, social class, etc.). This indicates that the newly graduated nursing students in general felt well prepared. However, five years in their profession this was almost the same, now ranging from 3.59 (for how prepared they were concerning equal rights from an LGBTQIA + perspective) to 5.70 (to collaborate with others).

There were some scores that differed significantly between the newly graduated nursing students and then five years in their profession. These concerned aspects on how prepared they were regarding working for equality, equitable treatment based on ethnic background, religion, social class, etc., equal rights from an LGBTQIA + perspective, and equitable treatment for people with disabilities. All these decreased with a mean of approximately 1 point on the Likert scale. However, only equal rights from an LGBTQIA + perspective scored below 4 five years in their profession, that could be considered low. Further, also the aspects regarding abilities to critically analyze information and to find relevant/necessary information decreased significantly five years in their profession. Nevertheless, the mean scores were still above 4.0, thus considered good (scores from 4 to 6). All the scores are presented in Table 2.

In summary, at the time of graduation and five years after their graduation, newly graduated nursing students and nurses perceived that they had knowledge to find relevant or necessary information, to critically analyze information, even though they were significantly lower five years in their profession. They also perceived that they had knowledge, skills/abilities, and tools to practice nursing in all aspects and to collaborate. Except for English they also felt well prepared to communicate verbally and in writing in Swedish.

**Table 2:** Distribution of responses from newly graduated nursing students (53/163) as well as after five years in their profession (32/163), from Karolinska Institutet, Sweden, regarding professional knowledge

	At graduation mean= (SD)	After 5 years in profession mean= (SD)	*p*-value
**I feel well prepared for my future profession needs**:			
To communicate in writing	5.08 (1.37)	5.13 (1.01)	0.501
To communicate verbally	5.26 (0.923)	5.43 (0.774)	0.827
To communicate in English	3.66 (1.87)	3.77 (1.38)	0.496
To critically analyze information	5.08 (1.37)*	4.67 (1.40)	0.021
To find relevant/necessary information	5.51 (0.639)*	4.10 (1.40)	0.001
To practice evidence based	5.15 (1.10)	5.10 (1.08)	0.907
To follow the development in my profession	4.70 (1.12)	4.83 (1.02)	0.728
To practice in my profession	5.00 (0.960)	4.53 (1.22)	0.323
To practice independently	5.13 (0.856)	5.27 (0.980)	0.962
To apply practical skills	4.96 (1.11)	4.75 (1.30)	0.104
To independently solve problems	5.04 (0.784)	5.40 (0.770)	0.197
To promote sustainable development	3.87 (1.87)	3.97 (1.33)	0.053
To collaborate with others	5.30 (0.774)	5.70 (0.794)	0.129
To collaborate with other professions	5.43 (0.665)	5.06 (0.982)	0.056
To work in interprofessional teams	5.29 (0.893)	5.10 (1.09)	0.107
To handle ethical considerations	5.13 (0.810)	4.47 (1.41)	0.473
To collaborate in multicultural environments	4.77 (1.37)	5.10 (1.09)	0.563
To work for equality	5.19 (1.21)*	4.19 (1.40)	0.001
Equitable treatment based on ethnic background, religion, social class, etc.	5.45 (0.992)*	4.47 (1.41)	0.001
Equal rights from an LGBTQIA + perspective	4.96 (1.49)*	3.59 (1.88)	0.003
Equitable treatment of people with disabilities	5.15 (1.39)*	4.00 (1.71)	0.01

* = Indicates significant difference (*p* < 0.05; Wilcoxon-test)

### Comparison of the perceived knowledge between newly graduated students from both professions

The newly graduated students from both professions scored in general high in all aspects of the degree evaluation. However, the newly graduated dental students mean (SD) scores were significantly higher than the newly graduated nursing students (5.31 (0.32) versus 5.00 (0.46)).

The newly graduated students in both professions perceived that they had knowledge in communication, in written and in verbal form in Swedish at the time of their graduation. There was no significant difference found between the programs. However, there was a significant difference between the professions concerning perceived knowledge to communicate in written and verbal form in English, where the dental students perceived their knowledge higher compared to the nursing students.

Also, both newly graduated dental and nursing students perceived that they had knowledge to handle ethical considerations, collaborate in multicultural environments, work for equality, equal rights from an LGBTQ + perspective, equitable treatment of people with disabilities and to give equitable treatment based on ethnic background, religion, social class by graduation based on the answers from the degree evaluation, as shown in Table 3.

There was no significant difference in the degree evaluation between the two professions concerning how the newly graduated students perceived knowledge to critically analyze information, to find relevant or necessary information, to practice evidence-based, and to follow the development in their profession. Also, there was no significant difference in the students’ perceived knowledge to practice in their profession, to practice independently, to apply practical skills, to independently solve problems and, to promote sustainable development. Furthermore, there was no significant difference between the two professions concerning perceived knowledge to collaborate with others, to collaborate with other professions and to work in interprofessional teams (Table 3).

**Table 3:** Distribution of responses mean (SD) from newly graduated dental students (23/71) and newly graduated nursing students (53/163) from Karolinska Institutet, Sweden, regarding professional knowledge

	Newly graduated dental students mean= (SD)	Newly graduated nursing students mean= (SD)	*p*-value
**I feel well prepared for my future profession needs**:			
To communicate in writing	5.52 (0.73)	5.08 (1.37)	0.253
To communicate verbally	5.57 (0.728)	5.26 (0.923)	0.719
To communicate in English	5.22 (1.17)*	3.66 (1.87)	0.022
To critically analyze information	5.61 (0.656)	5.08 (1.37)	0.493
To find relevant/necessary information	5.57 (0.59)	5.51 (0.639)	0.830
To practice evidence based	5.48 (0.665)	5.15 (1.10)	0.518
To follow the development in my profession	5.13 (0.968)	4.70 (1.20)	0.284
To practice in my profession	4.48 (1.31)	5.00 (0.960)	0.256
To practice independently	5.39 (0.839)	5.13 (0.856)	0.686
To apply practical skills	5.35 (0.647)	4.96 (1.11)	0.693
To independently solve problems	5.17 (0.887)	5.04 (0.784)	1.000
To promote sustainable development	4.70 (1.40)	3.87 (1.87)	0.182
To collaborate with others	5.61 (0.656)	5.30 (0.774)	0.292
To collaborate with other professions	5.09 (1.12)	5.43 (0.665)	0.192
To work in interprofessional teams	4.83 (1.47)	5.29 (0.893)	0.333
To handle ethical considerations	5.48 (0.593)	5.13 (0.810)	0.205
To collaborate in multicultural environments	5.22 (1.20)	4.77 (1.37)	0.412
To work for equality	5.36 (1.36)	5.19 (1.21)	0.716
Equitable treatment based on ethnic background, religion, social class, etc.	5.74 (0.541)	5.45 (0.992)	1.000
Equal rights from an LGBTQIA + perspective	5.43 (1.41)	4.96 (1.49)	0.444
Equitable treatment of people with disabilities	5.52 (1.31)	5.15 (1.39)	0.341

* = Indicates significant difference (*p* < 0.05; Wilcoxon-test)

### Comparison of the perceived knowledge between dental and nursing professionals five years in their profession

Both dentists and nurses five years in their profession scored in general high in all aspects of the alum evaluation, without any significant difference between the two professions with mean (SD) scores for dentists versus nurses were 4.85 (0.81) and 4.70 (0.59) respectively.

Both dentists and nurses perceived that they had knowledge in communication, in written and in verbal form in Swedish at the time of their graduation. There was no significant difference found between the programs. However, in both professions there was an expression of uncertainty concerning communication in written and verbal form in English, but also concerning equitable treatment of people with disabilities, and equal rights from an LGBTQIA + perspective. All these reached mean scores below 4.

Further, there was a significant difference between the two professions when it came to finding relevant/necessary information and to follow the development within their professions. Here the dentists expressed a higher degree of knowledge even though the nurses scored over 4 in mean indicating sufficient knowledge.

However, both dentists and nurses perceived that they had knowledge to handle ethical considerations, collaborate in multicultural environments, work for equality, and to provide equitable treatment based on ethnic background, religion, social class by graduation based on the answers from the alum evaluation, as shown in Table 4.

Finally, there was no significant difference regarding dentists and nurses perceived knowledge to practice in their profession, to practice independently, to apply practical skills, to independently solve problems and, to promote sustainable development. Furthermore, there was no significant difference between the two professions concerning perceived knowledge to collaborate with others, to collaborate with other professions and to work in interprofessional teams (Table 4).

**Table 4:** Distribution of responses from graduated dental students (12/71) and nursing students (32/163) five year in their profession, from Karolinska Institutet, Sweden, regarding professional knowledge

	Dentists 5 years in their profession mean= (SD)	Nurses 5 years in their profession mean= (SD)	*p*-value
**I feel well prepared for my future profession needs**:			
To communicate in writing	5.00 (1.21)	5.13 (1.01)	1.000
To communicate verbally	5.58 (0.669)	5.43 (0.774)	0.588
To communicate in English	3.75 (1.14)	3.77 (1.38)	0.056
To critically analyze information	6.00 (0.9)	4.67 (1.40)	0.281
To find relevant/necessary information	5.17 (1.34)*	4.10 (1.40)	0.014
To practice evidence based	5.75 (0.452)	5.10 (1.08)	0.086
To follow the development in my profession	5.00 (1.21)*	4.83 (1.02)	0.031
To practice in my profession	5.00 (0.996)	4.53 (1.22)	0.676
To practice independently	4.18 (1.47)	5.27 (0.980)	0.341
To apply practical skills	5.58 (0.669)	4.75 (1.30)	0.066
To independently solve problems	5.50 (0.674)	5.40 (0.770)	0.149
To promote sustainable development	4.50 (1.62)	3.97 (1.33)	0.525
To collaborate with others	5.67 (0.651)	5.70 (0.794)	0.371
To collaborate with other professions	4.67 (0.985)	5.06 (0.982)	0.200
To work in interprofessional teams	5.42 (0.793)	5.10 (1.09)	0.346
To handle ethical considerations	5.50 (0.674)	4.47 (1.41)	0.305
To collaborate in multicultural environments	5.00 (1.21)	5.10 (1.09)	0.892
To work for equality	3.92 (1.73)	4.19 (1.40)	0.408
Equitable treatment based on ethnic background, religion, social class, etc.	4.00 (2.09)	4.47 (1.41)	0.546
Equal rights from an LGBTQIA + perspective	3.08 (2.43)	3.59 (1.88)	0.234
Equitable treatment of people with disabilities	3.67 (2.32)	4.00 (1.71)	0.832

* = Indicates significant difference (*p* < 0.05; Wilcoxon-test)

## Discussion

### Confidence trends, literacy imbalances and professional challenges

The main findings of the study indicate a high level of confidence in both professions at graduation. However, five years into the profession, there was a noticeable decline in areas concerning equality and equitable treatment. Additionally, dentists reported a decrease in areas concerning communication in English, while nurses consistently reported low confidence regarding sustainable development and communication in English in both evaluations.

Both educational programs require English for admission and include at least one full course delivered in English, encompassing communication, lectures, literature, and examinations. However, these courses are not during clinical practice. In the dental program, students write, peer-review, and defend their degree projects in English, whereas nursing students primarily use Swedish for their degree projects. Furthermore, dental students are allocated double the time (30 ECTS), compared to the nursing students (15 ECTS) for completing these degree projects, creating an imbalance in opportunities to develop academic literacy, professional literacy, and professional knowledge. This imbalance reflects the distinct roles of academic literacy and professional literacy in shaping professional knowledge.

Academic literacy equips students with critical reading, writing, and communication skills required for academic success, while professional literacy focuses on practical, field-specific communication, judgment, and ethical practice, as noted in the introduction. Together, these literacies support the development of a well-rounded professional capable of applying theoretical knowledge in real-world settings [6–8]. In addition to an extended time for degree projects, dental students benefit from regular English training throughout their program, with an intensive period at the end, likely enhancing their confidence in English communication [30, 31]. However, five years after graduation, this confidence may decline due to less structured professional needs for English communication, in contrast to nurses, whose English exposure during education aligns more closely with professional requirements.

### Program structures and their impact on literacy and professional competence

The duration of these programs – five years for the dental education and three years for the nursing education – implies that dental students have more time to develop and connect academic and professional literacies with professional knowing. Nursing students, by comparison, may have fewer opportunities for such integration, including less time for practical application and structured guidance and support from faculty. Affordances – opportunities perceived within an environment [32] – are presented differently in these two programs. In professional education, instructors use varied teaching strategies to create learning affordances. In line with previous studies, nursing students may lack exposure to the systemic value of evidence-based practice within their profession, making professional knowledge feel more abstract and disconnected from real-world contexts [17–19, 33, 34]. This disconnection can hinder the seamless integration of academic literacy, professional literacy, and professional knowledge, which are foundational for critical thinking and informed decision-making in practice. These elements, as emphasized in the introduction, are essential for healthcare practitioners navigating complex, interdisciplinary healthcare roles [6–11].

The time allocated in education has significant implications, as demonstrated by responses from the evaluations. Dentists scored higher than nurses in most aspects at graduation; however, five years later, dentists scored lower than nurses in aspects related to equality and equitability. This shift underscores the critical role of professional literacy in fostering equity within healthcare settings. Professional literacy, as defined earlier, involves mastering inclusive communication styles, understanding cultural nuances, and respecting diverse patient backgrounds – competencies that are integral to delivering equitable and effective care. These skills are not only a fundamental part of professional knowledge but are also reinforced through critical thinking, a literacy-based competency essential for ethical and informed practice. Inclusive communication and cultural competence are particularly important within professional literacy, as they enable practitioners to navigate the diverse social and cultural contexts of healthcare. When students are trained to integrate inclusion into teamwork and uphold human rights, they develop literacy skills that enhance patient communication and promote fairness. These competencies strengthen professional knowledge by helping students address diverse patient needs, mitigate potential biases, and make well-informed decisions in their practice [35]. Furthermore, Swedish healthcare training ensures that future professionals are prepared to deliver fair, high-quality care to diverse patient populations. This commitment to equity supports continuous professional development and encourages a holistic approach to addressing patient needs, thereby fostering a more ethical and responsive healthcare environment [35–37].

Another structural difference is dental students receive pre-clinical training, simulation practice (on virtual patients/phantom heads), and clinical training with real patients *in-house*, that is, within a university dental clinic (https://ki.se/en/udc/university-dental-clinic-at-karolinska-institutet). Nursing students, in contrast, have pre-clinical training in centers with simulation equipment, and perform clinical practice in various healthcare settings outside the university. Research shows that the learning environment is crucial for professional development, with regular supervision and continuity of teaching contributing to stronger learning outcomes [38–40].

### Translating literacy skills into professional practice

The items in the evaluation – such as written and verbal communication, critical analysis, evidence-based practice, and interprofessional collaboration – represent essential literacy skills directly tied to professional knowledge in nursing and dentistry. These competencies are not standalone but are deeply interconnected with the broader development of academic and professional literacies, forming the foundation for effective practice. In educational settings, skills like communication and critical analysis are introduced through coursework, assignments, and clinical simulations, preparing students for documentation, team-based problem-solving, and ethical decision-making [41, 42]. Evidence-based practice, for example, relies on critical analysis and information literacy, equipping students to assess and apply research in clinical care. As emphasized in the introduction, these literacy skills extend beyond technical abilities, reflecting the socio-cultural and situational contexts of healthcare [3]. Academic literacy prepares students for rigorous documentation and research, while professional literacy enables them to adapt and apply these skills in patient-centered and team-based environments. This dual focus bridges theoretical education with practical application, ensuring that professional knowledge is both contextually relevant and effectively applied in in real-world healthcare settings [6–8].

While both programs aim to develop these competencies, real-world application depends on the consistency and reinforcement of these skills during education. Dental students benefit from in-house clinical training and structured feedback, reinforcing literacy skills within a familiar environment, whereas nursing students face more diverse clinical environments that demand adaptability, but sometimes lack consistent supervision. This variation affects their confidence in applying literacy skills across different contexts, highlighting the impact of the learning environment on professional readiness. Thus, while both professions receive foundational literacy training, the translation to practice is influenced by educational affordances and real-world challenges, affecting long-term confidence and role effectiveness [43, 44]. Thus, as previously noted, the alignment of academic and professional literacy with professional knowledge is not only a pedagogical challenge but also a critical determinant of students’ ability to transition from education to practice effectively.

Consequently, dental students have more opportunities and ongoing supervision with calibrated teachers who guide their development [30, 31]. Nursing students, supervised by clinical instructors who vary with each clinical setting, lack this continuity, affecting their development. Conversely, nursing students experience real-world situations sooner, but without the smooth transition provided by the consistent environment that dental students have [38, 45]. This experience may impact nursing students’ confidence in academic literacy, professional literacy, and professional knowledge [30]. Although, nursing students gain experience integrating these areas [25, 26, 46], limited affordances during their education can make it challenging to clearly identify the value of each skill for the profession. For students to make use of these educational opportunities, they must recognize them – whether in academic literacy, professional literacy and professional knowledge – and be capable of transforming them into effective practice [32]. Capacity in this sense may derive from the meaningfulness of the learning content, which in turn motivates students [47, 48]. Developing a strong professional identity depends on this connection. Without a clear professional link, the content’s significance may be diminished, giving dental students an advantage [38].

### Study strengths and limitations

A strength of this study is its inclusion of two professional higher education programs that are both similar and distinct in meaningful ways. These programs were chosen because they share common ground in emphasizing the importance of professional and academic literacy, particularly in preparing students to communicate effectively, make informed decisions, and engage in evidence-based practice. Both professions require a high degree of interpersonal interaction and scientific decision-making, which ties closely to the competencies developed through academic and professional literacy.

However, the programs also differ in significant ways that make this comparison valuable. For example, the study program in dentistry is longer, at an advanced level, and focuses deeply on specialized areas within medicine, while the study program in nursing is shorter, at a basic level, and encompasses a broader scope of healthcare practice. These differences allow us to explore how the distinct structures, levels, and educational frameworks of the two programs shape the development and application of literacy skills in professional practice.

By comparing these study programs, the purpose was to highlight how literacy is cultivated and utilized across different types of healthcare education. This approach also underscores the shared importance of communication, evidence-based decision-making, and patient-centered care, while providing insights into how these competencies are emphasized and developed differently within each educational context. This dual perspective offers valuable insights into the role of literacy in shaping professional readiness in distinct but interconnected healthcare fields.

However, there are also some limitations to address. When it comes to the decrease in confidence over time regarding professional knowledge, this finding does not necessarily indicate that there is an actual lack of competence, but it could rather be a consequence of a more realistic understanding of the complexities of practice. However, the methodology and present data cannot answer this, which could be considered a limitation of this study. Another limitation is the low response rate from dentists and nurses at the time of graduation, compounded by an even lower response rate five years post-graduation which limits the representativeness of the findings. Consequently, the results may not accurately reflect the opinions or perceptions of all newly graduated dentists and nurses, but the ones of the participants involved in this study. Thus, the results should not be generalized to the broader populations of these professional groups. Nevertheless, despite these limitations, the study provides valuable insights into the likely trends and directions.

## Conclusion

The level of confidence and perception of a sufficient degree of knowledge regarding academic literacy, professional literacy, and professional knowledge is higher at the time of graduation in both professions compared to five years into the profession, where there is a decrease in areas concerning work in relation to equitability, and equality, and for dentists also communication in English. Furthermore, nurses had a constant low confidence rating in both evaluations concerning sustainable development and communication in English. This indicates that targeted and continuous professional development is crucial to address these challenges and to bridge the gap between the knowledge and confidence levels at graduation and the evolving demands of professional practice over time. Thus, when reconstructing the overall curriculum in professional educations it is of great importance to provide tools to enhance future professional development rather than the perception of that they can rely solely on their education at graduation time.

## Data Availability

The raw material is available from the corresponding author on reasonable request.
